# Assessment of safety during hospitalization for patients undergoing Surgery after neoadjuvant therapy for moderately advanced Esophageal cancer

**DOI:** 10.1186/s12893-023-02252-8

**Published:** 2023-11-08

**Authors:** Chenyi Xiong, Hongyun Ji, Feng Li, Zhihua Jiang, Zhonghao Pang, Xiaoran Li

**Affiliations:** 1 Department of Thoracic Surgery, Affiliated Hospital of Jiangsu University, Zhenjiang, Jiangsu 212000 China; 2 Institute of Digestive Diseases, Affiliated Hospital of Jiangsu University, Zhenjiang, Jiangsu 212000 China

**Keywords:** Esophageal cancer neoadjuvant therapy security assessment retrospective study

## Abstract

**Purpose:**

To study the safety of patients with moderately advanced esophageal cancer during their hospital stay after undergoing surgery.

**Methods:**

The clinical and pathological data of 66 patients with locally advanced esophageal cancer discharged from the Department of Thoracic Surgery of Jiangsu University Hospital from January 2017 to October 2022 were selected, of whom 32 underwent direct surgery (control group) and 34 underwent neoadjuvant therapy followed by surgery (experimental group), to retrospectively analyze whether there were differences in surgical outcomes, complication rates, biochemical and infection indicators between the two groups.

**Results:**

The number of lymph node dissections, lymph node dissection rate, and hemoglobin value on the first day after the operation in the experimental group were smaller than those in the control group, and the difference was statistically significant (P < 0.05). The thoracic drainage volume of the experimental group was more than that of the control group, and the difference was statistically significant (P < 0.05). The incidence of pulmonary complications in the experimental group was higher than that in the control group, especially pulmonary infection, and the difference was statistically significant (P < 0.05). Compared with the control group, the experimental group was more prone to anastomotic leakage, and the difference was statistically significant (P < 0.05).

**Conclusion:**

Neoadjuvant therapy combined with surgery for patients with advanced esophageal cancer is generally safe during hospitalization.

## Introduction

Esophageal cancer is a common digestive tract tumor, which is the seventh largest malignant tumor in the world, and its mortality rate ranks sixth among malignant tumors in the world [1]. China is a high-incidence area of esophageal cancer and one of the countries with the highest mortality rate of esophageal cancer. Different from the type of esophageal cancer in European and American countries, the pathological type of esophageal cancer in China is mainly squamous cell carcinoma, which may be caused by different economic levels, ethnic differences, dietary habits, and gene mutation types [2]. However, due to the insidious onset of esophageal cancer, it will not have clinical manifestations such as dysphagia until the middle and late stages. About 70% of patients are already in the local advanced stage before the pathological diagnosis is confirmed [3]. Relying on simple surgical treatment cannot achieve satisfactory results, and its 5-year survival rate is less than 30% [4, 5]. The NCCN guidelines recommend neoadjuvant therapy as the first-line treatment for patients with advanced esophageal cancer [6]. NEOCRTEC 501 study has pointed out that neoadjuvant chemotherapy has certain advantages for esophageal squamous cell carcinoma and can improve the surgical resectability rate of patients with esophageal cancer [7]. The commonly used neoadjuvant chemotherapy regimen in China is the treatment regimen of platinum combined with paclitaxel [8]. However, neoadjuvant therapy also has certain complications for patients themselves. Some studies have pointed out that the risk of complications such as respiratory failure and anastomotic leakage of esophageal cancer after neoadjuvant therapy will increase [9]. Whether we should give up the comprehensive treatment of advanced esophageal cancer to avoid the potential risks of neoadjuvant therapy? Will this not be worth the loss? This article retrospectively collected the relevant data of 66 patients with locally advanced esophageal cancer who occurred in Zhenjiang, a high-incidence area of esophageal cancer in China, to illustrate the safety of neoadjuvant therapy for patients with locally advanced esophageal cancer.

## Materials and methods

### Patients

Clinical and pathological data of 66 patients with locally intermediate and advanced esophageal cancer discharged from the Department of Thoracic Surgery, Affiliated Hospital of Jiangsu University from January 2017 to October 2022 were systematically collected retrospectively through inpatient cases, and the tumor location and clinical stage of patients were collected according to the *China Esophageal Cancer Diagnostic and Treatment Standard (2018 version)*. There were 66 patients in the whole group, 32 cases received direct surgery (control group) and 34 cases underwent neoadjuvant therapy followed by surgery (experimental group). In the control group, there were 26 males and 6 females, aged 51–82 years, with a mean age of 65.9 years; in the experimental group, there were 31 males and 3 females, aged 49–74 years, with a mean age of 62.7 years. There was no statistically significant difference in age and gender between the two groups (P > 0.05).

Inclusion criteria for the neoadjuvant therapy followed by surgery group: (1) preoperative gastroscopic pathology report confirmed the diagnosis of esophageal malignancy (squamous carcinoma); (2) relevant adjuvant examinations judged that patients could tolerate neoadjuvant therapy; (3) patients met the neoadjuvant therapy guidelines according to the *China Esophageal Cancer Diagnostic and Treatment Standard (2018 version)*. (4) The patient had no history of other malignancies and no distant metastasis of esophageal malignancies. (5) At 4–6 weeks after receiving neoadjuvant therapy.

Inclusion criteria for the direct surgery group: (1) patients refused to receive neoadjuvant therapy; (2) preoperative gastroscopic pathology report confirmed the diagnosis of esophageal malignancy (squamous carcinoma); (3) clinical stage at stage III was confirmed by adjuvant means such as gastroscopy, endoscopic ultrasonography, and PET-CT according to the *China Esophageal Cancer Diagnostic and Treatment Standard (2018 version)*; (4) relevant adjuvant tests (liver and kidney function, blood routine, lung function test, enhanced CT, etc.) to determine that the patient could tolerate surgical treatment; (5) the patient had no history of other malignant tumors.

### Treatment modality

#### Neoadjuvant therapy

According to the actual situation of our hospital, in this trial, neoadjuvant patients were treated with a regimen of paclitaxel 135 mg/m^2^ + nedaplatin 60 mg/ m^2^ (intravenous infusion), which was repeated every three weeks. Four to six weeks after the end of treatment, a multidisciplinary consultation was conducted to comprehensively assess the patient’s physical condition and determine whether the surgery could be tolerated through blood routine, liver and kidney function, upper gastrointestinal tract imaging, enhanced CT and PET-CT.

#### Preparation for Surgery

Patients in the experimental and control groups were fasted from the afternoon of the day before the operation and were given polyethylene glycol electrolytes to eliminate intestinal contents as prescribed by the doctor, and half the usual dose of a second-generation cephalosporin (cefonicid sodium 1 g) was given intravenously half an hour before the operation as a prophylactic medication.

#### Surgical approach

According to the *China Esophageal Cancer Diagnostic and Treatment Standard (2018 version)*, the preoperative examination should be improved and the preoperative preparation should be done. According to the diagnosis and treatment standard, the mass is 15–20 cm from the incisor for upper segment esophageal cancer, 20–25 cm from the incisor for middle segment esophageal cancer, and 25–30 cm from the incisor for lower segment esophageal cancer. Patients with middle and upper esophageal cancer were operated by Mckeown and patients with lower esophageal cancer were operated by Ivor-Lewis, and systemic lymph node dissection was performed in two or three fields according to the lymph node enlargement observed intraoperatively, and postoperative gastric tube, nasogastric tube and chest drain were left in place.

### Observed indexes

The operating time, intraoperative bleeding, gastrointestinal tube retention time, chest drainage, number of intraoperative lymph node dissection, number of lymph node metastases, length of hospital stay, complication rate, white blood cell count, hemoglobin, C-reactive protein and albumin on the first postoperative day were recorded.

### Statistical analysis

Data were analyzed by SPSS software version 26.0. The measurement data with normal distribution were expressed as mean ± standard deviation and the t-test was used for comparison between groups. The Chi-square test or Fisher’s exact test was used for comparison between the two groups. P < 0.05 was considered statistically significant.

## Results

### General case information

There was no significant difference in gender, age, tumor location, and clinical stage (P > 0.05) (Table 1).

**Table 1:** Comparison of general data between two groups of patients [case(%)]

Parameter	Experimental group	Control group	P-value
	(n = 34)	(n = 32)	
Sex			0.240
Male	31	26	
Female	3	6	
Age	62.7(49–74)	65.9(51–82)	0.090
Tumor location(%)			0.738
Upper	4(11.8)	2(6.3)	
Middle	16(47.1)	16(50.0)	
Lower	14(41.1)	14(43.7)	
Pathological staging			0.378
IIIA	7(20.6)	4(12.5)	
IIIB	27(79.4)	28(87.5)	

### Operative results

In both groups, there were no deaths during hospitalization and postoperative pathology confirmed the complete removal of the mass, and all procedures were performed by the same four surgeons. To determine whether the anastomosis and tubular stomach were bleeding after surgery and to reduce postoperative tubular gastric reflux that could lead to aspiration, we placed a gastric tube during the surgery and to maintain the patient’s nutritional status, we gave an intraoperative jejunal tube to ensure postoperative intrajejunal nutrition. The number of intraoperative cleared lymph node metastases confirmed in the postoperative pathology report was defined as the number of postoperative lymph node metastases, and the number of intraoperative lymph node cleared and the number of postoperative cleared lymph node metastases in the experimental group were less than those in the control group, and the difference was statistically significant (P = 0.019, P = 0.032), and the chest drainage in the experimental group was more than that in the control group, and the difference was statistically significant (P = 0.044) while the two groups of patients The differences in operative time, intraoperative bleeding, gastrointestinal tube placement time and hospital stay were not statistically significant (P > 0.05) (Table 2).

**Table 2:** Comparison of surgical results between two groups [case(%)]

Parameter	Experimental group	Control group	P-value
	(n = 34)	(n = 32)	
Operation time	285.1 ± 72.5	291.0 ± 54.9	0.713
Intraoperative blood loss (ml)	188.2 ± 151.3	135.9 ± 173.3	0.196
Pleural effusion(ml)	2512.0 ± 1745.0	1711.0 ± 1391.0	0.044
Number of lymph node dissection(s) Number of postoperative lymph node metastasis (s)	19.3 ± 10.4 1.2 ± 2.0	24.9 ± 8.4 2.1 ± 1.3	0.019 0.032
Gastric tube placement time (days)	10.1 ± 4.1	8.7 ± 3.1	0.128
Nasointestinal tube placement time (days)	18.2 ± 21.0	13.0 ± 10.1	0.153
Hospitalization time (days)	29.9 ± 23.9	24.2 ± 1.9	0.323

### Postoperative complication

In this data collection, the incidence of respiratory complications was higher in the experimental group compared to the control group, with a statistically significant difference (P = 0.001), especially pulmonary infections (P = 0.012), and the incidence of the anastomotic fistula was greater in the experimental group than in the control group, with a statistically significant difference (P = 0.030). The remaining indicators in the two groups were not statistically significant (P > 0.05).The other indicators were not statistically significant (P > 0.05) (Table 3).

**Table 3:** Comparison of postoperative complications between two groups of patients [cases (%)]

Operative complications	Experimental group	Control group	P-value
	(n = 34)	(n = 32)	
Respiratory system	22(64.7)	7(21.9)	0.001
pulmonary infection^a^	12	3	0.012
Pneumothorax	1	0	0.328
Respiratory failure^b^	9	4	0.154
Circulatory system	5(14.7)	4(12.5)	0.794
Cardiac insufficiency	3	3	0.905
Arrhythmia	2	1	0.591
Digestive system	7(20.6)	1(3.1)	0.030
Anastomotic leakage^c^	7	1	

^a^postoperative chest X-ray or CT shows the presence of new or progressive infiltrative, solid and ground glass shadows with a temperature greater than 38 °C

^b^patients with an arterial partial pressure of oxygen less than 60 mmHg with or without a partial ^c^pressure of carbon dioxide greater than 50 mmHg in the resting state, breathing air

evaluation by upper gastrointestinal imaging (iodine) on the sixth postoperative day

### Infection indicators and biochemical indicators on the first postoperative day

In this study, leukocyte count and C-reactive protein on the first postoperative day were selected as indicators of infection, and hemoglobin and albumin on the first postoperative day were used as biochemical indicators for observation. The mean hemoglobin of the experimental group was lower than that of the control group and statistically significant (P = 0.032), while the leukocyte count and C-reactive protein of the experimental group were higher and the albumin was lower than that of the control group, but the difference was not statistically significant (P > 0.05) (Table 4).

**Table 4:** Infection indicators and biochemical indicators on the first postoperative day

Parameter	Experimental group	Control group	P-value
	(n = 34)	(n = 32)	
Hemoglobin(g/L)	116.4 ± 16.7	125.1 ± 13.7	0.032
White blood cell count (10^9^/L)	15.3 ± 5.4	13.4 ± 3.5	0.105
C-reaction protein (mg/L)	80.7 ± 45.7	66.2 ± 40.4	0.198
Albumin (g/L)	33.7 ± 3.1	33.8 ± 3.8	0.942

## Discussion

The incidence of Esophageal malignant tumors is high in China and their onset is insidious, so most patients are already in the middle and late stages when they are diagnosed. Although surgery is still the first choice for patients with esophageal cancer who have indications for surgery, their 5-year survival rate is less than 20% because they often have lymph node metastases [4]. This suggests that a single treatment option is not beneficial for patients to prolong their life span. In recent years, with the continuous development of neoadjuvant therapies, an integrated treatment plan with surgery, radiotherapy, and immunotherapy is gaining more and more attention from clinicians. The CROSS multicenter randomized controlled phase III study in the Netherlands noted that patients receiving neoadjuvant therapy had a higher 5-year survival rate compared to patients undergoing direct surgery [10], and similar conclusions were reached in the OEO2 study in Japan [11]. Neoadjuvant therapy has taken an irreplaceable place in the comprehensive treatment regimen for intermediate to advanced esophageal cancer.

In this study, the number of lymph nodes cleared intraoperatively and the number of lymph nodes confirmed to have metastases postoperatively were less in patients who had undergone neoadjuvant therapy than in the control group, indicating that neoadjuvant therapy can achieve a stage-reducing effect for patients with intermediate to advanced esophageal cancer, thereby reducing the chance of postoperative metastases and prolonging the survival of patients. Since esophageal cancer surgery is a digestive tract reconstruction surgery, there are many contents containing bacteria in the intestine, so intestinal preparation and early postoperative enteral nutrition are particularly important. Early enteral nutrition can not only promote the early recovery of intestinal function and meet the nutritional needs of patients but also accelerate the recovery of intestinal immune function and reduce the incidence of intestinal infection [12, 13]. According to the actual clinical practice of our hospital after the operation, we will give the input of enteral nutrient solution through the indwelling gastrointestinal tube on the day after the operation, and routinely apply the second-generation cephalosporin (cefonicid sodium 2 g) with sufficient measurement to prevent the corresponding infection. Antibiotics are used until the patient is discharged from the hospital. However, even so, due to the overall impact of surgery or neoadjuvant therapy on patients, including the decline of patients ' immune function or the change of tissue blood supply and local microenvironment near the anastomosis, these often lead to postoperative complications of esophageal cancer [14]. In terms of postoperative complications, patients who have received neoadjuvant therapy are more likely to have pulmonary complications, especially pulmonary infections, which may be due to the fact that neoadjuvant therapy is an immune shock and physical exertion for patients, which easily impairs cellular immune function and reduces the recognition ability and immune response ability of lymphocytes to pathogens [11], and it has also been pointed out that neoadjuvant therapy, especially for patients who have undergone radiotherapy, has a significant impact on the survival of patients. It has also been suggested that patients undergoing radiotherapy are prone to pulmonary fibrosis after radiotherapy, which can cause radiation pneumonia and is related to the dosage of radiation [15], which can lead to poor basal lung function and postoperative sputum weakness, resulting in pneumonia.

The most typical symptom of patients with mid to late-stage esophageal cancer is progressive dysphagia, which causes malnutrition and aggravates the tumor burden due to tumor encroachment on the esophagus, making it compressed or even difficult to eat. For patients after neoadjuvant therapy, their digestive tract response and immunosuppression will be heavier, and malnutrition has become an independent risk factor for poor prognosis of patients with esophageal cancer [15]. In this trial, the hemoglobin level in the experimental group was significantly lower than that in the control group, and the difference was statistically significant, while the difference in albumin, which is usually used as an indicator to assess the nutritional status of patients, was not significant. The possible reason for this is mainly that the index selected for this experiment was the first day after surgery. For patients after neoadjuvant therapy, due to its side effects, it tends to lead to bone marrow suppression as well as increased body metabolism, causing complications such as anemia and hypoproteinemia, which are usually treated accordingly by clinicians, such as blood transfusion and human albumin input, etc. Wait until 4–6 weeks after the end of neoadjuvant therapy, and then the thoracic surgeon to assess the feasibility of surgery. As hemoglobin has a longer half-life than albumin, it takes longer to recover than albumin and is more difficult to recover from. Among the nutritional complications of esophageal cancer, anastomotic fistula is a more serious complication, occurring in around 10% of cases and increasing perioperative mortality [16], especially in patients who have received neoadjuvant therapy, which can lead to histological damage such as ischaemic changes caused by local vasoconstriction [17].

In summary, although there will be complications during the hospitalization of neoadjuvant therapy combined with surgery for advanced esophageal cancer, most of them are within the clinically controllable range, which is generally safe and feasible. However, because this experiment is a single-center, retrospective case analysis, the data sample size is small, and there will inevitably be some bias. But we can see a certain trend, and get some general direction. These still need a large sample, multi-center, prospective studies to further explore and analyze.

## Data Availability

The raw data of this paper are available upon reasonable request to the corresponding author.
